# Quantum theory of mass potentials

**DOI:** 10.1371/journal.pone.0198929

**Published:** 2018-07-05

**Authors:** Dmitriy Melkonian, Terry Blumenthal, Edward Barin

**Affiliations:** 1 Faculty of Medicine and Health Sciences, Macquarie University, Sydney, Australia; 2 Department of Psychology, Wake Forest University, Winston-Salem, United States of America; University of Minnesota, UNITED STATES

## Abstract

Probabilistic formalism of quantum mechanics is used to quantitatively link the global scale mass potential with the underlying electrical activity of excitable cells. Previous approaches implemented methods of classical physics to reconstruct the mass potential in terms of explicit physical models of participating cells and the volume conductor. However, the multiplicity of cellular processes with extremely intricate mixtures of deterministic and random factors prevents the creation of consistent biophysical parameter sets. To avoid the uncertainty inherent in physical attributes of cell ensembles, we undertake here a radical departure from deterministic equations of classical physics, instead applying the probabilistic reasoning of quantum mechanics. Crucial steps include: (1) the relocation of the elementary bioelectric sources from a cellular to a molecular level; (2) the creation of microscale particle models in terms of a non-homogenous birth-and-death process. To link the microscale processes with macroscale potentials, time-frequency analysis was applied for estimation of the empirical characteristic functions for component waveforms of electroencephalogram (EEG), eye-blink electromyogram (EMG), and electrocardiogram (ECG). We describe universal models for the amplitude spectra and phase functions of functional components of mass potentials. The corresponding time domain relationships disclose the dynamics of mass potential components as limit distribution functions produced by specific microscale transients. The probabilistic laws governing the microscale machinery, founded on an empirical basis, are presented. Computer simulations of particle populations with time dependent transition probabilities reveal that hidden deterministic chaos underlies development of the components of mass potentials. We label this kind of behaviour “transient deterministic chaos”.

## Introduction

Physiological mass potentials produced as a result of electrochemical activity of excitable cells are noninvasive, reliable, and objective markers of various psychophysiological functions. EEG, eye blink EMG, and ECG, typical examples of mass potentials, have been used in a wide variety of research and clinical applications in humans, to study basic stimulus processing, attentional factors, emotion, personality variables, dysfunction in clinical populations, etc. However, the interpretation of mass potentials rests mainly on an empirical understanding, and so an adequate theory of the underlying generation mechanisms would be of great value.

Phenomenologically, the mass potential is a product of hierarchically organized physiological systems with multiple levels of organization, from molecular to cellular [1]. The attempts to create quantitative relationships between the global and cellular scales in electrophysiology are known as the forward and inverse problems, respectively, particularly in electroencephalography [2] and electrocardiography [3]. Thus far, these widely researched problems have been studied using methods of classical physics. The assumption common to all approaches is that elementary voltages produced in some way by the underlying cells are “building blocks”, the linear summation of which creates the mass potential. This linear model means that a global scale potential contains parameters of all participating microscopic scale sources of electricity. This leads to intractably huge numbers of degrees of freedom and prevents a unique determination of the mass effect.

The current study is the first, to our knowledge, to propose a radically different approach to the theory of mass potentials, based on the probabilistic formalism of quantum mechanics. A fundamental aspect of quantum mechanics that is not present in classical physics is the statistical nature of its assertions. In classical mechanics, the state of a system uniquely determines the values of all the physical quantities associated with it. In quantum mechanics, the state of a system defines the physical quantities only as random variables, i.e., it determines the laws of distributions obeyed by the physical quantities.

Statistical methods of quantum mechanics have been successfully applied to describe the *macroscopic picture* emerging in many-particle systems with a host of *microscopic random effects*. A classic example is Brownian motion, which portrays the macroscopic picture emerging from erratic movements of tiny particles suspended in a fluid [4]. Numerous physical applications include gases, fluids, semiconductors, plasma, electrons and ions in conductors, etc.

Each application necessitates development of a specific probabilistic model. Particularly significant revisions of the theory and computational methods are needed to deal with biological objects. A crucial step in this context is the relocation of elementary charges underlying generation of mass potentials from the cellular level to the molecular level. Accordingly, elementary sources of electricity in our theory are charged particles, the ions which cross the cell membrane in both directions. The size and stochasticity of these entities conform their attributes to quantum physics.

The probabilistic nature of ion transport was discovered by the patch-clamp technique, which provided a means of measuring ion currents through individual ion channels in the cellular membrane [5]. A fundamental finding has been that individual ion channels are essentially stochastic entities that open and close in a random way. A probabilistic interpretation of this evidence implies that the channel state may be treated as a memoryless random variable [6]. This means that the future state of the channel depends only on its present state, and not on how that state was reached. The remarkable consistency of these properties with assumptions of the Markov processes inspired the development of stochastic models of ion channels using continuous-time discrete-state Markov chains [7]. The major challenge is that Markov chains are hidden in the time course of observable processes.

Empirically based approaches to overcome this difficulty include a group of techniques known collectively as hidden Markov models [8–10]. A critical challenge is to reconcile stochastic mechanisms of single ion channels with deterministic behavior of observable processes. A new feature of our theory is that observable processes are components of mass potentials produced by huge numbers of charged particles acting on the microscopic scale. Thus, we deal not with the details of ion channel gating nor the chemical nature of ions but with the sizes of extracellular charged particle ensembles produced by trans-membrane transport processes.

This problem was addressed in part in the context of quantal transmitter turnover [11–12]. The Markov model was formulated in terms of the birth and death process (BDP). Having adopted this probabilistic framework, we consider a component waveform as a transient potential, and construct its microscale model using BDP of a “transient” type. The time course of this non-homogenous BDP is defined by the time dependent birth and death rates that are not known in advance.

Our goal was to identify these entities by using only experimentally determined components of various mass potentials (EEG, ECG, and eye-blink EMG) so that testable predictions of emergent global scale behaviour have no element of circularity.

To solve this problem we have developed an original methodology of time-frequency analysis using the similar basis function (SBF) algorithm. The methodology is presented in the Materials and Methods section **(**see **Time-frequency analysis using the SBF algorithm).** In the same section we present an algorithm of numerical simulations which provide the means to compare empirically based solutions with the theory (see **Algorithm of numerical simulations**).

In the Results section we present the following outcomes of our study:

- A novel microscale model of ion transport created in terms of a non-homogenous BDP. On this basis we introduce an integral equation linking micro- and macro- scale processes.
- Empirical identification of universal models of amplitude spectra and phase functions of half-waves from EEG, ECG, and eye-blink EMG records. On this basis we deduce the characteristic function and half wave function (HWF) as universal the frequency and time domain constituents of mass potentials.
- Introduction of nonlinear macroscale equations as the tools for description of the dynamics of mass potentials.
- Identification of the primary and secondary particle populations and formulation of the probabilistic rules that govern transport of particles in these populations during resting and transient conditions.
- Numerical simulations of the micro scale processes, which reveal transient amalgamation of deterministic and stochastic trends, with this process termed transient deterministic chaos.
- The statement of the self-similarity of HWFs indicating the universality of the theory.

In the discussion section we point out that probabilistic formalism of our theory provides solutions to problems unresolved by previous deterministic models.

## Results

### Ion transport in terms of nonhomogeneous BDP

Mass potentials resemble electrical phenomena occurring in ensembles of multiple excitable cells immersed in an interstitial fluid. The elementary bioelectric sources acting at the microscopic scale are ions, both positively and negatively charged particles, which cross the cell membrane in both directions. The membrane serves as a separating material that divides the tissue into the extracellular and intracellular compartments. Due to a high resistance, relative to the resistance of the extracellular space, the cell membranes are rather good electrical insulators [13]. This prevents the charged particles located inside the cells from producing measurable changes in potential differences between various spatial locations in the extracellular space. Based on this evidence we presume in our theory that potential fields in the extracellular space are produced by extracellular charges, i.e. the extracellular particle populations.

Since ion channels capture and release ions in a random way, the pertinent extracellular particle population develops as a stochastic process. It is generally accepted that Markov processes with discrete states in continuous time are adequate mathematical models for stochastic interpretation of ion-channel mechanisms [7].

A specific aspect of our approach is that we deal not with the details of ion channel gating but with extracellular particle populations produced by the ion transport processes. We consider the cell membrane as a border that separates extracellular particle populations from particles inside the cells. Through this border an ion enters or leaves the extracellular particle population. To describe these events we adapt the theoretical framework of the previous modelling studies of the short-term synaptic plasticity in which the BDP, an important class of Markov processes, has been settled as a tool for modelling particle populations of neurotransmitters [11–12].

Let the integer-valued time-dependent random variable **X**(t) (here and throughout the paper boldface letters denote random variables) measure at time t the size of the population of charged particles (ions acting as point charges) involved in the creation of an extracellular monopole. Following conventional notions we regard the number of particles x_*i*_ = **X**(*t*_*i*_) as the state of the particle population at the time instant t_i_. The chances of inter-state transitions are evaluated by the transition probabilities expressed in terms of the birth and death rates.

We implement the main assumption of the Markov process, that during a sufficiently small element of time, Δ, the probability of the change of the **X**(t) by more than one particle is negligibly small:

P[X(t+Δ)=X(t)+k]=ο(Δ)if|k|>1, (1)

where P denotes probability and k is an integer.

Therefore, the particle system can change its state only through transition to the nearest neighbours. An increase of the population size by a unit represents birth, **X**(t+Δ) = **X**(t)+1, whereas a decrease by a unit represents death, **X**(t+Δ) = **X**(t)-1. Thus, a particle moving out of a cell would constitute a ‘death’ for the inside of the cell and a ‘birth’ for the extracellular compartment. Wide classes of BDPs with constant transition probabilities deal with stationary processes. However, the complex dynamics of global scale potentials indicates the transient changes in behavior of the underlying particle systems. Thus we employ the BDP of a “transient” type. As the most suitable mathematical tool we use the nonhomogeneous BDP in which the birth and death rates may be any specified functions of the time t [14]. The probabilities of population changes for nonhomogeneous BDP are:

P[X(t+Δ)=X(t)+1]=X(t)⋅Δ⋅λ(t)+ο(Δ),(birth) (2)

P[X(t+Δ)=X(t)−1]=X(t)⋅Δ⋅μ(t)+ο(Δ),(death) (3)

where λ(t) and μ(t) and are the birth and death rates, respectively.

Let us choose Δ in compliance with Eq (1) and represent the time evolution of **X**(t) as the succession of discrete states x_i_. The permitted states of the particle population at the time t_i+1_ are: x_i+1_ = x_i_+1 (birth), x_i+1_ = x_i_ (unchanged size), or x_i+1 =_ x_i_-1 (death). The probabilities of the corresponding inter-state transitions are:

$$P[x_{i+1}=x_{i}+1]=\hat{p}(i)+ο(\Delta ),\;(birth)$$

$$P[x_{i+1}=x_{i\;}-1]=\overset{ˇ}{p}(i)+ο(\Delta ),\;(death)$$

where $\hat{p}(i)$ and $\overset{ˇ}{p}(i)$ denote the time dependent transition probabilities for birth and death, respectively. From Eqs (2) and (3) we get:

$$\hat{p}\left(i\right)=x_{i}\cdot \Delta \cdot \lambda \left(t_{i}\right),\;\;\left(\text{birth}\right)$$ (4)

$$\overset{ˇ}{\text{p}}\left(i\right)=x_{i}\cdot \Delta \cdot \mu \left(t_{i}\right),\;\;\left(\text{death}\right)$$ (5)

These equations provide the means for a step-by-step evaluation of the time evolution of the particle population. However, the functions λ(t) and μ(t) are not known in advance. Our goal is to estimate these functions on an empirical basis. Thus, we need to link the changes of the particle population acting on the microscopic scale with observable dynamics of the global scale processes.

To outline a physical basis for such an approach we suppose that transport of particles creates a thin cloud of positive and negative ions spread over the outer surfaces of the cell membranes. Particle distribution that takes place uniformly over the membrane surface would not change the resting conditions of the extracellular current flow and would therefore generate no extracellular potential transients. The event that disturbs the uniformity of particle distributions and creates measurable potential changes in the extracellular space acting as a monopole is a transient synchronization of ion flows across cellular membranes of large ensembles of functionally linked excitable cells. Such a time-locked transient in the particle population is induced by a triggering event and consequently can be specified by the time instant from which the process started.

Taking t = 0 as the beginning of the transient, the expected trajectory of the mean population size e(t) from 0 to t is given by [14]

e(t)=E{X(t)}=exp[-ρ(t)],

where E{} denotes expected value and

$$\rho (t)=\int_{0}^{t}[\mu (\xi )-\lambda (\xi )]d\xi .$$

Combining the above equations we obtain

$$\text{e}\left(t\right)=\text{exp}\left\{-\int_{0}^{t}\left[\mu \left(\xi \right)-\lambda \left(\xi \right)\right]d\xi \right\}$$ (6)

According to the theory of the field potentials within the frequency range of physiological interest, the extracellular space may be regarded with reasonable accuracy as a resistive medium [13, 15]. Thus, we may assume that the voltage of the global scale transient potential u(t) is proportional to the expected population size, i.e. u(t) = k∙e(t), where k is the coefficient of proportionality. We regard integral equation Eq (6) as a bridge linking extracellular potentials with the variables λ(t) and μ(t) governing the microscopic scale processes.

### Gaussian amplitude spectrum of empirical half-wave

We assume that a transient increase of the particle population size is reflected on the global scale by peaking waveforms of relevant mass potentials. Such an approach is in harmony with the widely accepted notion that various positive and negative peaks (maxima and minima) in the waveform of the recorded potential reflect the summed activity of the underlying cellular populations with specific structure-function organization.

To identify peaking waveforms in the time course of a mass potential we need to estimate segments over which the waveform deflections develop. The methodology adopted here is that of the method of fragmentary decomposition introduced in earlier works [16, 17].

Considering a mass potential as the time function v(t), we deal with a series of recorded values v_*m*_ = v(*t*_*m*_) at regular, discrete time intervals *t*_*m*_ = *m*Δ, where Δ is the sampling interval. The segmentation points are defined as zero-crossings and points of global and local minima of |v(t)|. More particularly, if (v_*m*−1_ ≤ 0 AND v_*m*+1_ > 0) OR (v_*m*−1_ ≥ 0 AND v_*m*+1_ < 0), then τ_i_ = t_m_ is qualified as zero-crossing (segmentation point), where i is the number of the segmentation point. The point τ_i_ = t_m_ specifies a global or local minimum of |v(t)| if |v_m-1_|≥|v_m_|≤|v_m+1_|. In this way a sequence of segmentation points τ_0_,…,τ_i_,…,τ_N_ is formed.

The signal segment between adjacent segmentation points is regarded as an empirical half wave (HW). Given a segment of the length T_*i*_ = *τ*_*i*_ − *τ*_*i*+1_ between the points τ_i-1_ and τ_i_ (i = 1,…,N), the HW is defined as

$${\text{w}}_{i}\left(t\right)=\{\begin{array}{ll} \text{v}\left(t+\tau_{i-1}\right) & \text{if}\;0\leq t\leq T_{i},\\ 0 & \text{otherwise}. \end{array}$$

In the interval from 0 to T_i_ this function reproduces the fragment of the signal v(t) between adjacent segmentation points τ_i-1_ and τ_i_. Therefore, in the interval [τ_0_, τ_N_]

$$\text{v}\left(t\right)=\sum_{i=1}^{N}{\text{w}}_{\text{i}}\left(t-\tau_{i}\right).$$ (7)

Suppose that the empirical HW incorporated by this equation is produced by microscale processes considered in the previous section and summarized by Eq (6). If this is the case, we expect that the relationships between the micro- and macro- scales should be indicated by some specific features of eligible HWs.

To approach this problem we combine consideration of the time domain on the one hand, and the frequency domain on the other, using the time-frequency analysis, which yields more powerful results than does considering the two domains separately.

The major procedure of the time-frequency analysis of a mass potential v(t) is the transformation of each w_i_(t) to the frequency domain. The frequency domain counterpart of the w_i_(t) given in the [0, T_i_] interval is defined by the exponential finite Fourier transform

$${\text{W}}_{i}\left(i\omega \right)=\int_{0}^{T_{i}}{\text{w}}_{\text{i}}\left(t\right)\text{exp}\left(-i\omega t\right)dt,$$ (8)

where ω = 2πf, f is frequency and $i=\sqrt{-1}$.

A significant computational challenge is that readily available techniques of digital spectral analysis, such as the Fast Fourier transform, are not suited to short-term spectral decompositions. As an adequate tool of time-frequency analysis we use the SBF algorithm [18] (see Materials and methods: **Time-frequency analysis using the SBF algorithm** for details).

**Fig 1:**
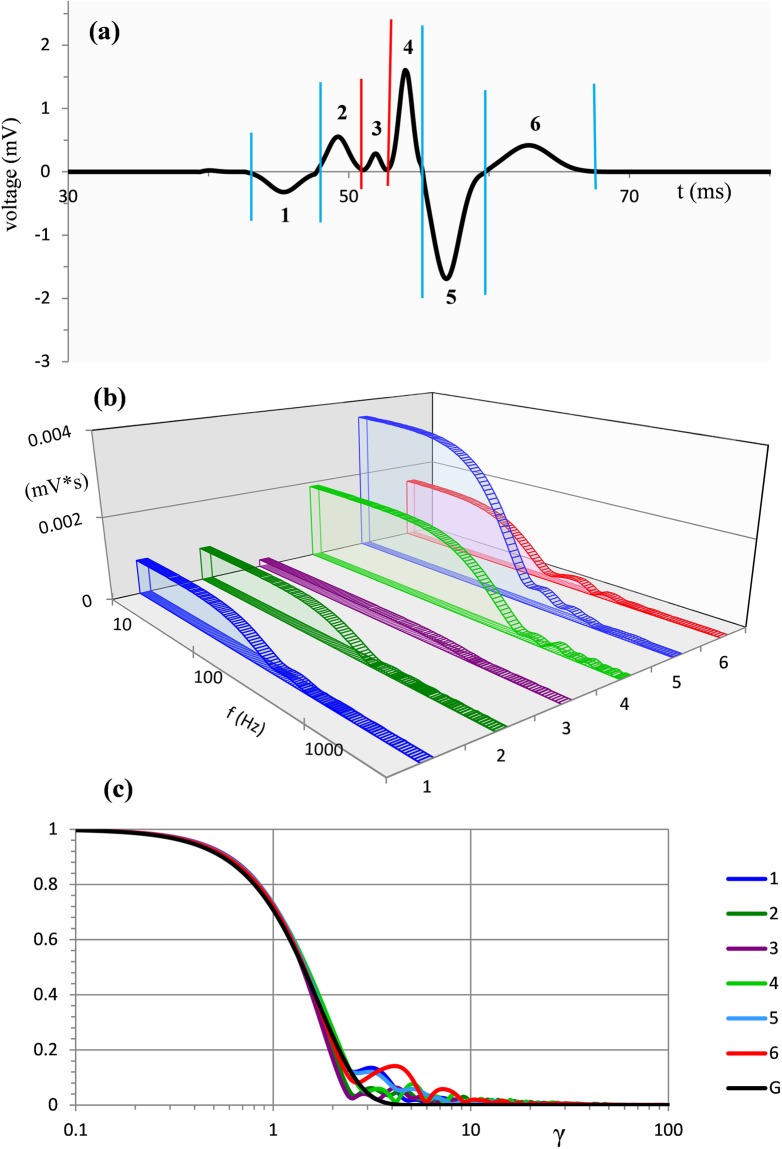
Typical eye-blink EMG in the time and frequency domains. (a) Record of single trial eye-blink EMG in the time interval starting 30 ms after stimulus onset. Vertical lines indicate segmentation points. (b) Amplitude spectra of component waveforms 1–6 from (a). (c) Coloured lines are the amplitude spectra from (b) after normalization of both amplitude and frequency. The black line shows the Gaussian function G(γ).

Typical results of the time-frequency analysis are presented in Fig 1. The curve in Fig 1a is a single trial eye-blink EMG (see Materials and methods: **Empirical mass potentials: EEG, ECG, eye-blink EMG** for details). Vertical blue and red lines indicate zero-crossings and minima (absolute values), respectively. These segmentation points separate 6 half waves in the time course of the record.

Using a logarithmic scale with 100 samples per decade, computed amplitude spectra of the corresponding half waves are shown in Fig 1b. To emphasize a strong similarity in the profiles of various amplitude spectra we transform the spectra into a universal dimensionless form using normalization of both the amplitude and frequency.

For normalization of the amplitude we chose a sufficiently small value ω_0_ to satisfy the condition: W(ω_0_)≈W(0). The normalized amplitude spectrum is defined as W*(ω) = W(ω)/W(ω_0_). This transition from physical to relative units is accounted for by considering W(ω_0_) as a scaling coefficient κ, i.e. κ = W(ω_0_).

**Fig 2:**
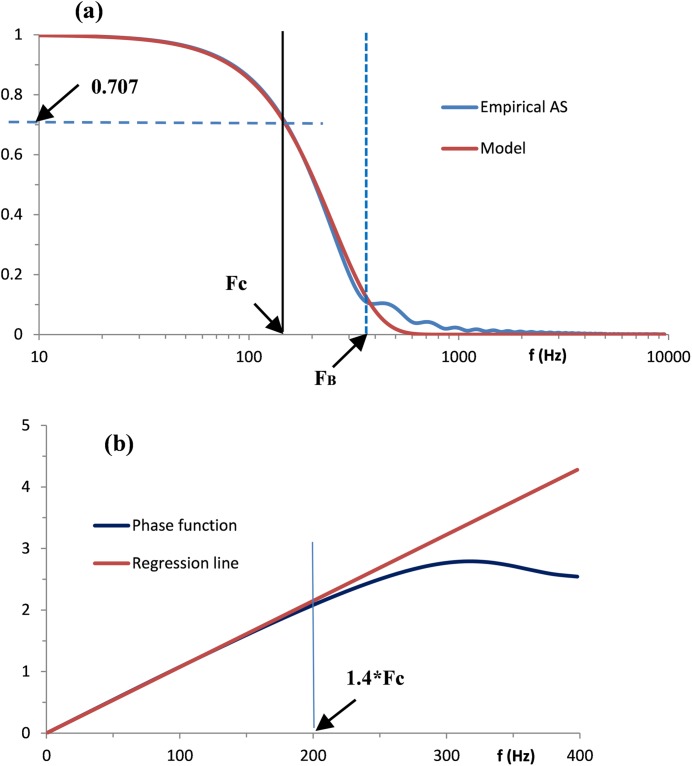
Empirical frequency characteristics computed by the SBF algorithm from component waveform 5 in Fig 1a and their models. (a) Amplitude spectrum and Gaussian model. The arrows show cut-off frequency F_C_ and boundary frequency F_B_. The y-axis units are dimensionless. (b) Phase function and linear regression line. The arrow indicates the right boundary point of the frequency range over which the deviation of the phase function from linearity is neglected. The y-axis units are radians.

Using HW 5, for example, the normalized amplitude spectrum is depicted as a blue line in Fig 2a.

This is a typical form of W*(ω) for the analytical model of which we use the Gaussian spectrum suggested as the frequency domain model of the amplitude spectra of the event related potential (ERP) and eye-blink EMG components in previous papers [16, 19]. Thus, we consider an expected profile of empirical W*(ω) in the form of the Gaussian function

$$\text{G}\left(\omega \right)=\text{exp}\left[-{\left(\sigma \omega \right)}^{2}/2\right],$$ (9)

where σ is a parameter.

We may regard W*(ω) as the frequency response of a low pass filter, a conventional parameter of which is the cut-off frequency F_C_. At this frequency the attenuation of the amplitude spectrum drops by 3dB, i.e. W*(ω_C_) = 1/√2 (≈0.707), where ω_C_ = 2πF_C_. Given F_C_, $\sigma =\sqrt{\text{ln2}}/2\pi {\text{F}}_{\text{c}}.$

For the empirical amplitude spectra in Fig 2a, F_c_ = 148 Hz. Combining W*(ω) and G(ω) at this frequency, we obtain an excellent fit of the Gaussian spectrum from Eq (9) to the normalized empirical amplitude spectrum.

For normalization of the frequency scale we use F_C_ as a basis unit and define dimensionless relative frequency γ = ω/2πF_C_ = ω/ω_C_. Appropriately scaled in magnitude and frequency, the standard empirical amplitude spectrum is defined as Z(γ) = W*(ω_c_γ).

Using (9) as the model of W*(ω), an expected form of Z(γ) is the Gaussian spectrum defined as

$$\text{G}(\gamma )=\text{exp}(-\gamma^{2}).$$

Note that Z(γ) = G(γ) at γ = 1, the relative frequency which corresponds to f = F_C_. Amplitude spectra from Fig 1b transformed to this dimensionless form are plotted in Fig 1c.

**Fig 3:**
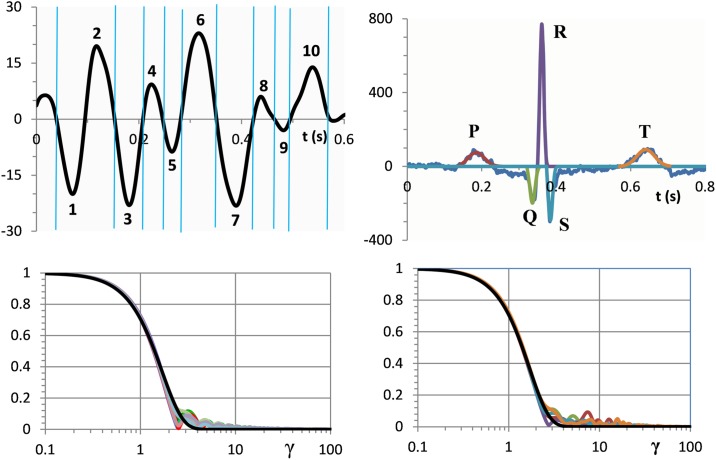
Upper panel: EEG (on the left side) and ECG (on the right side) records selected for the time-frequency analysis (the y-axis units are μV). 10 half-waves selected for EEG analysis represent signal fragments between segmentation points indicated by vertical lines. 5 selected ECG half-waves correspond to the waveforms of P, Q, R, S and T components. Lower panel: normalized amplitude spectra (coloured lines) in comparison with Gaussian curves (black lines). The y-axis units are dimensionless.

In a similar way we have applied the time-frequency analysis to the HWs from records of other mass potentials. The advantage of the frequency domain is that multiple forms of HWs are reduced to a universal simple analytical form of Gaussian model G(γ). Fig 3 illustrates typical results for EEG and ECG records shown in the upper panel. Superposed dimensionless amplitude spectra are shown in the lower panel. These and numerous similar analyses of different HWs from various EEG, ECG, and eye-blink EMG recordings indicate that combining Z(*γ*) and G(*γ*) at γ = 1 produces remarkably accurate fits of analytical G(γ) to empirical Z(*γ*).

To estimate accuracy of fits we define the dimensionless extension ratio ε = F_B_/F_C_, where F_B_ is the boundary frequency, i.e. the frequency below which the mean square error (MSE) between W*(ω) and G(ω) does not exceed the threshold value T. The fit in Fig 2a, with ε = 1.74, gives a visual idea of how F_C_ and F_B_ are estimated.

According to our theory, the global scale components of mass potentials reflect statistical distributions of underlying elementary sources acting on the microscopic scale. We may interpret the amplitude spectra as global scale averages of statistical distributions.

Because we deal with amplitude spectra of EEG, ECG, and EMG signals originated from functionally different sources, an important problem was to determine whether the statistical distributions produced by the corresponding samples are different or indistinguishable in statistical terms. The parameter ε is an adequate tool for this purpose because this single parameter is sufficient to evaluate the fitting results.

We estimated values for different HWs from varieties of EEG, ECG, and eye-blink EMG records (see Materials and methods: **Kolmogorov Smirnov test** for details). The HW was accepted as eligible for further analysis if the number of the HW samples was ≥8. This condition eliminated from analysis a small portion of HWs, approximately 2–3%.

Using datasets of ε dimensionless parameters collected from different sources we applied the two-sample Kolmogorov-Smirnov test to examine the null hypothesis that the samples are drawn from populations with the same distribution. The test has the advantage of making no assumption about the particular distribution of data, i.e. it is non-parametric and distribution free. To apply the tests we created separate samples from EEG, EMG, and ECG data, each containing values of ε from 100 different HWs. The means and standard deviations (SD) of ε values were as follows. EEG: mean = 1.781 (SD = 0.215); ECG: mean = 1.792 (SD = 0.187); eye-blink EMG: mean = 1.788 (SD = 0.227).

**Fig 4:**
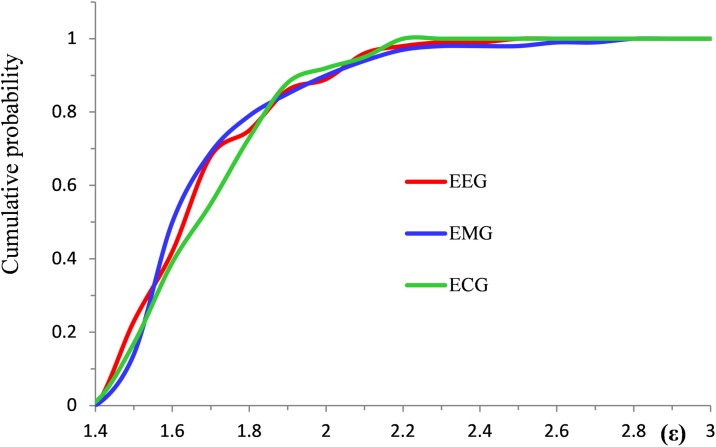
Kolmogorov-Smirnov statistical test applications for typical EEG, eye-blink EMG, and ECG data. Red, blue, and green lines are empirical cumulative distribution functions.

Normalized by the sample size, empirical cumulative distribution functions evaluated from each of these sample sets are illustrated in Fig 4: EEG—red line, ECG—green line, and eye-blink EMG—blue line.

Given two cumulative distribution functions normalized by the sample size, the greatest discrepancy between these measures, called the D-statistic, serves as a criterion to reject the null hypothesis. Given equal size for both samples (100 values of ε in the tests in question), the null hypothesis is rejected if D≥0.2 (5%).

The maximum absolute differences computed for different pairs are as follows. EEG vs ECG: 0.13; EEG vs EMG: 0.09; ECG vs EMG: 0.14. Similar results documented for empirical half waves from various records are well below 0.2 and do not provide any reasons to reject the null hypothesis. It is important to note the highly stereotypical character of the test results repeated for various HWs.

### Linear phase function

The calculations of the amplitude spectra and F_B_ parameter were followed by the estimation of the phase functions. We found that the initial part of the empirical phase function δ(ω) shows consistency with a simple linear model

φ(ω)=βω, (10)

where β is a parameter.

As numerous calculated linear fits indicate, the deviation from linearity can be neglected over the frequency range from 0 to 1.4∙F_C_. The typical result is illustrated in Fig 2b. Thus, the estimation of β was reduced to the calculation of linear regression lines using δ(f) samples from f = f_0_ to f = 1.4∙F_C_. The slope of the regression line serves as the estimate of β.

### Characteristic and half-wave functions

Deduced analytical dependencies in Eqs (9) and (10) define a universal equation of the complex spectrum in the form

$$\text{G}\left(i\omega \right)=\text{exp}\left\{-\left[{\left(\sigma \omega \right)}^{2}/2\right]-i\beta \omega \right\}$$ (11)

Referring to the probabilistic formalism of quantum mechanics, we qualify complex value G(iω) as a characteristic function [20]. Accordingly, the time domain counterpart of the characteristic function, g(t), can be considered as a distribution.

A conventional relationship between the characteristic function F(iω) and the corresponding time domain distribution function f(t) is established by the reciprocal Fourier integrals:

$$\text{f}(t)=\frac{1}{2\pi }\int_{-\infty }^{\infty }\text{F}(i\omega )\text{exp}(i\omega t)d\omega ,$$

$$\text{F}(i\omega )=\int_{-\infty }^{\infty }\text{f}(t)\text{exp}(-i\omega t)dt.$$

If F(iω) in the first of these integrals is expressed over an infinite frequency scale by the Eq (11), i.e. F(iω) = G(iω), then f(t) is a normal distribution defined on an infinite time scale. However, we deal with a causal process which starts from the time instant when the triggering event initiates the component development. Such a condition establishes specific relationships between the real and imaginary parts of G(iω). Both functions contain the same information, and either one alone is sufficient to find the time domain counterpart [21]. We refer to the imaginary part

$${\text{G}}_{I}(\omega )=Im[\text{G}(i\omega )]=\text{exp}[-{\left(\sigma \omega \right)}^{2}/2]\text{sin}(\beta \omega ).$$

Using this function we define the time domain solution at t>0 by the sine Fourier transform of G_I_(ω)

$$\psi (t)=\frac{2}{\pi }\int_{0}^{\infty }\text{exp}[-[{\left(\sigma \omega \right)}^{2}/2]]\text{sin}(\beta \omega )\text{sin}(\omega t)d\omega .$$

This integral has an analytical solution [22]. For t≥0

$$\psi \left(t\right)={\left(\sigma \sqrt{2\pi }\right)}^{-1}\left[\psi_{\text{P}}\left(t\right)-\psi_{\text{S}}\left(t\right)\right],$$ (12)

where

$$\begin{array}{l} \psi_{P}\left(t\right)=\text{exp}\left[-{\left(t-\beta \right)}^{2}/2\sigma^{2}\right],\\ \psi_{S}\left(t\right)=\text{exp}\left[-{\left(t+\beta \right)}^{2}/2\sigma^{2}\right]. \end{array}$$

**Fig 5:**
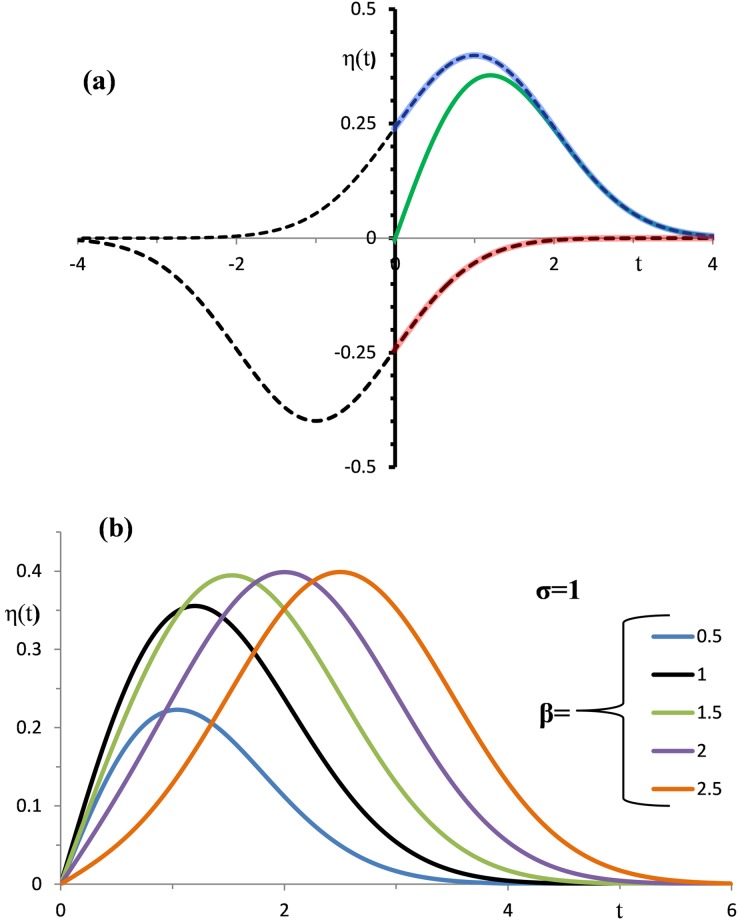
Half-wave function and its components. (a) The green, blue, and red solid lines show η(t), η_P_(t), and η_S_(t) with σ = β = 1 parameters. The dotted lines are Gaussian functions which disclose η_P_(t) and η_S_(t) as fragments of the two shifted curves of normal distributions. (b) Effects of varying β on the shapes of η(t) with σ = 1.

These functions are illustrated in Fig 5a by the solid lines: ψ(t)—green, ψ_P_(t)—blue and ψ_S_(t)–red. The dotted lines indicate that ψ_P_(t) and ψ_S_(t) are the fragments of shifted normal distributions.

Eq (12) is consistent with the *wave function* in a general form of the d’Alembert’s solution [23]. A crucial difference is that the latter is defined on an infinite time scale while ψ(t) defined by Eq (12) is zero at t<0. To take into account this specific feature we term ψ(t) a *half-wave function* (HWF).

### Nonlinear macroscale equations

The system producing HWF is expressed by the following system of nonlinear differential equations:

$$\begin{array}{l} d\left[\psi_{P}\left(t\right)\right]/dt=\left[-\left(\beta -t\right)/\sigma^{2}\right]\psi_{P}\left(t\right),\\ d\left[\psi_{S}\left(t\right)\right]/dt=\left[-\left(\beta +t\right)/\sigma^{2}\right]\psi_{S}\left(t\right),\\ \psi \left(t\right)=\psi_{P}\left(t\right)-\psi_{S}\left(t\right). \end{array}$$ (13)

We may qualify the system as a non-autonomous nonlinear system, that is, a system driven by time-varying processes.

Given σ = 1, coloured lines in Fig 5b show ψ(t) with different values of β (0.5, 1,1.5, 2, 2.5). Notable changes in the waveform profiles indicate that ψ(t) has the potential to fit empirical half-waves with various relationships between ascending and descending shapes.

### Rules of birth and death in particle populations

We wish to deduce ψ(t) as a product of the temporal evolution of a microscale particle population underlying component generation. We regard the appearance of the two terms in Eq (12) as indications of the two identifiable particle sub-populations, the *primary particle population* associated with ψ_p_(t) and the *secondary particle population* associated with ψ_s_(t).

We first consider the primary particle population, using as a model the BDP with the birth and death rates λ_P_(*t*) and μ_P_(*t*), respectively. Inserting e(t) = ψ_p_(t) in Eq (6) we obtain

$$\text{exp}\left\{-\int_{0}^{t}[\mu_{\text{P}}(\xi )-\lambda_{\text{P}}(\xi )]d\xi \right\}=\text{exp}[-{\left(t-\beta \right)}^{2}/2\sigma^{2}].$$

Consequently,

$$\int_{0}^{t}[\mu_{\text{P}}(\xi )-\lambda_{\text{P}}(\xi )]d\xi ={\left(t-\beta \right)}^{2}/2\sigma^{2}.$$

It is a simple matter to solve this equation and deduce

$$\lambda_{\text{P}}(t)=\lambda_{\text{P}}=\beta /\sigma^{2}\;\text{and}\;\mu_{\text{P}}(t)=t/\sigma^{2}.$$

Turning to the secondary particle population, we express ψ_s_(t) in terms of the birth and death rates denoted by λ_*S*_(*t*) and μ_S_(*t*), respectively. Replacement of e(t) in Eq (6) by ψ_s_(t) gives us

$$\text{exp}\left\{-\int_{0}^{t}[\mu_{\text{S}}(\xi )-\lambda_{\text{S}}(\xi )]d\xi \right\}=\text{exp}[-{\left(t+\beta \right)}^{2}/2\sigma^{2}]$$

Consequently,

$$\int_{0}^{t}[\mu_{\text{S}}(\xi )-\lambda_{\text{S}}(\xi )]d\xi ={\left(t+\beta \right)}^{2}/2\sigma^{2}.$$

It follows from the solution of this equation that

$$\lambda_{\text{S}}(t)=0\;\text{and}\;\mu_{\text{S}}(t)=\left(t+\beta \right)/\sigma^{2}=\lambda_{P}+\mu_{\text{P}}(t).$$

We summarize results in the form of the following rules governing the birth and death rates for the primary and secondary particle populations:

***Rule 1*.** *After activation at t = t*_*0*_ *by the triggering event*, *the transient behavior of the primary particle population develops as a non-homogenous BDP with the constant rate of birth*
  $$\lambda_{P}=\beta /\sigma^{2}.$$ (14)
  *and the time dependent rate of death*
  $$\mu_{\text{P}}\left(t\right)=\left(t-t_{0}\right)/\sigma^{2}.$$ (15)
***Rule 2*.** *After activation at t = t*_*0*_ *by the triggering event*, *the transient behavior of the secondary particle population develops as a non-homogenous death process with the time-dependent rate of death*
  $$\mu_{\text{S}}\left(t\right)=\left(t-t_{0}+\beta \right)/\sigma^{2}=\mu_{\text{P}}\left(t\right)+\lambda_{P}.$$ (16)

Turning to the resting conditions, it is essential that ion transport is balanced for both cations and anions. This means that, in the resting states, the sizes of the primary and secondary particle populations fluctuate over constant mean values. We consider the development of these events as simple BDPs with constant rates of birth and death. To define parameters of these processes we note that Eqs (14)–(16) contain the time dependent term μ_p_ (t) and the time independent term λ_p_. On a physical basis we associate the time dependent μ_p_(t) with the gated ion channels and constant λ_p_ with the resting ion channels. In this context λ_p_ participates in both the resting and transient conditions. Thus, we consider λ_p_ as a universal estimate for the birth and death rates during the resting conditions. Accordingly, the ion transport, balanced for both particle populations, is supported by the condition:

$$\lambda_{P}^{r}=\mu_{P}^{r}=\lambda_{S}^{r}=\mu_{S}^{r}=\beta /\sigma^{2},$$ (17)

where $\lambda_{P}^{r},$, $\mu_{P}^{r},$, $\lambda_{S}^{r}$ and $\mu_{S}^{r}$ denote the resting state rates of birth and death for primary and secondary particle populations.

It is important to note that resting state conditions are not recognizable from the global scale. The parameters included in Eq (17) are deductions from the rules governing the transient regimes. Thus we may expect the existence of additional resting state parameters which do not affect the transient components.

### Transition probabilities

Given identified σ and β parameters, established rules of birth and death allow us to complete, on an empirical basis, the description of transition probabilities (4) and (5). We deduce the following formulas of transition probabilities for transient conditions.

Primary particle population:

$${\hat{p}}_{p}\left(i\right)=x_{i}\cdot \Delta \cdot \beta /\sigma^{2},\;\;\left(\text{birth}\right)$$ (18)

$${\overset{ˇ}{p}}_{P}\left(i\right)=x_{i}\cdot \Delta^{2}\cdot i/\sigma^{2}.\;\;\left(\text{death}\right)$$ (19)

Secondary particle population:

$${\overset{ˇ}{p}}_{S}\left(i\right)=x_{i}\cdot \Delta \cdot \left(i\Delta +\beta \right)/\sigma^{2}.\;\;\left(\text{death}\right)$$ (20)

According to Eq (17) the birth and death processes in the primary and secondary particle populations are governed under the resting conditions by the birth and death rates equal to β/σ^2^. The corresponding transition probability is

$$p_{r}\left(i\right)=x_{i}\cdot \Delta \cdot \beta /\sigma^{2}.$$ (21)

Now we have an explicit set of equations for transition probabilities that provide means for numerical reconstructions of the time evolution of the particle populations during the transient and resting conditions.

### Physical basis of numerical simulations

**Fig 6:**
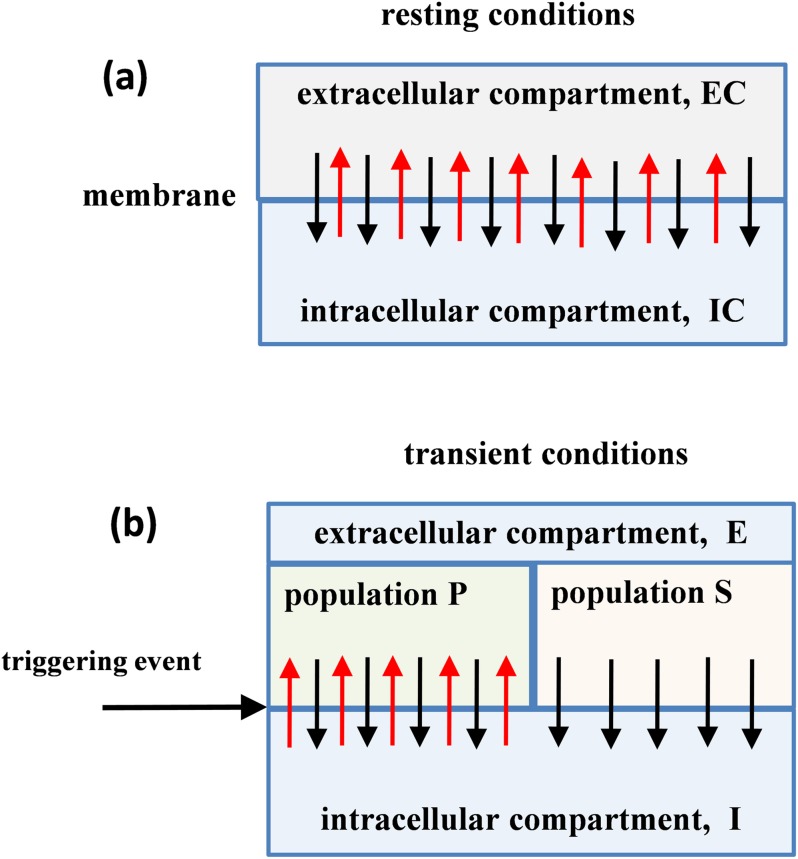
Block scheme of particle exchange between intra-cellular and extra-cellular ion compartments divided by a membrane. The black and red arrows show directions of the membrane crossings by ions of opposite polarity. (a) Resting conditions with balanced transmembrane ion transport. (b) Formation of primary (P) and secondary (S) extracellular particle populations during transient conditions.

Numerical simulations are unique tools used to reconstruct the time courses of particle populations in different trials. To provide a physical basis, the schemes in Fig 6 indicate significant aspects of the resting and transient conditions. A major element is the membrane which separates intracellular space from extracellular space. An ensemble of extracellular ions can be considered as a thin cloud of cations and anions spread over the outer surfaces of the cell membrane. During the resting condition illustrated in Fig 6a the transmembrane ion transport is balanced. According to Eq (17) the birth and death rates are equal for positive and negative particles. This means that, given a local volume, the numbers of positive and negative particles fluctuate over some mean values.

The transient conditions arise from induced synchronized activity in an ensemble of closely located and functionally linked excitable cells. According to our theory, such activity causes transient changes in the membrane machinery that controls the ion exchange between the intracellular and extracellular compartments. The most important effect identifiable by our theory, from the macroscopic scale, is the appearance of differences between the behavior of positive and negative charges. The scheme in Fig 6b shows the triggering event, the influence of which on the transmembrane ion transfers divides particles in the extracellular compartment into the primary (P) and secondary (S) populations, governed by different rules. A crucial effect following from Rule 2 above is the blockage of the “birth” processes in the secondary particle population. Thus, during the transient conditions the particles in the primary population behave as a non-homogenous birth and death process while the particles in the secondary population behave as a non-homogenous death process.

### Numerical simulations

The numerical simulations were designed to reconcile solutions of the system described by non-linear deterministic Eq (13) with the probabilistic model of non-homogenous BDPs describing the behaviour of individual particles in our theory. To deal with particle numbers we consider a numerical counterpart of Eq (12) in the form: **X**_N_(t) = **X**_P_(t)-**X**_S_(t), where **X**_P_(t) and **X**_S_(t) are the numbers of particles in the primary and secondary populations, respectively. **X**_N_(t) is the number of particles producing the net charge.

A general procedure is organized as the succession of standard steps dealing, in sequential order, with the temporal evolution of x_i_ over the time intervals [t_i_, t_i+1_] were i takes values from -M to N-1. The time interval from t_-M_ to t_0_ corresponds to the resting condition. At t = t_0_ the resting state is switched to the transient conditions calculated from t_0_ to t_N_ (see Materials and methods: **Algorithm of numerical simulations** for details).

**Fig 7:**
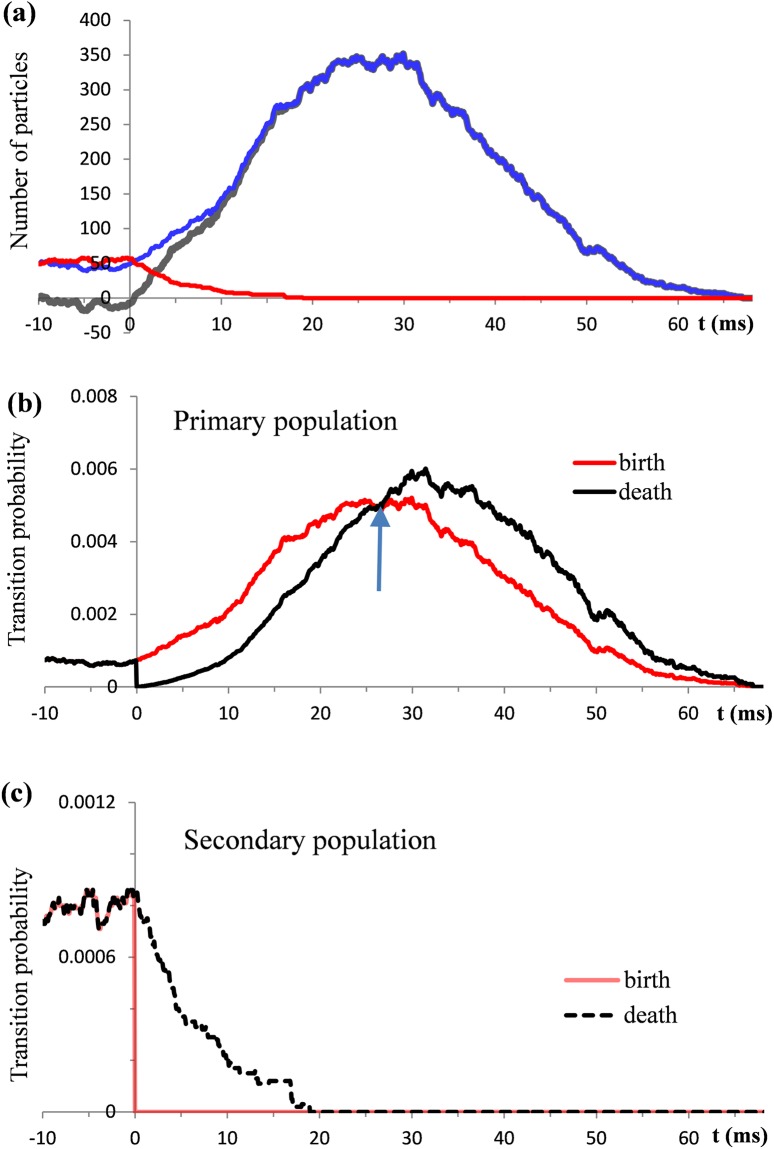
Numerical reconstructions of the temporal evolution of particle populations in a typical trial. Resting conditions computed from -10 ms are switched at t = 0 to the transient conditions. (a) Blue, red, and black lines show functions **X**_P_(*t*), **X**_S_(*t*) and **X**_N_(*t*), respectively. (b, c) Time courses of the underlying transition probabilities.

Simulations illustrated in Fig 7 extend over the time interval from -10 to 70 ms, with t = 0 corresponding to the instant at which the transient starts. As an initial condition, an equal size N_0_ = 50 was prescribed for both populations. The parameters σ = 13.3 ms and β = 26.2 ms were taken from Table 1 for EEG half-wave 2.

**Table 1:** Parameters of half-wave functions.

	HW	τ	κ	σ	β	F_C_	ε
		s	μV∙s	s	s	Hz	RU
**EMG**	1	0.043	-0.00078	0.00103	0.00245	129	1.74
	2	0.0476	0.000751	0.000728	0.00159	182	1.82
	3	0.051	0.000158	0.000409	0.00081	331	2.04
	4	0.0526	0.00172	0.00054	0.00141	246	2.69
	5	0.0552	-0.00314	0.000896	0.00174	148	1.74
	6	0.0594	0.00163	0.00149	0.00305	89	1.78
**EEG**	1	0.04	-0.619	0.0124	0.0286	10.7	1.78
	2	0.096	0.648	0.0133	0.0262	10	1.74
	3	0.156	0.822	0.0103	0.0210	12.9	1.78
	4	0.208	0.2	0.00856	0.0181	15.5	1.55
	5	0.248	-0.166	0.00762	0.0164	17.4	1.66
	6	0.284	0.897	0.0156	0.0327	8.51	1.62
	7	0.352	-0.923	0.0159	0.0346	8.31	1.66
	8	0.424	0.124	0.00836	0.016	15.8	2.34
	9	0.464	-0.0501	0.0068	0.016	19.5	1.7
	10	0.496	0.553	0.0159	0.04	8.32	1.82
**ECG**	P	0.136	3.99	0.0205	0.05	6.46	1.86
	Q	0.308	-3.91	0.0096	0.0251	13.8	1.66
	R	0.348	7.19	0.00504	0.0133	26.3	1.7
	S	0.375	-4.62	0.00836	0.0129	15.8	1.55
	T	0.58	5.04	0.023	0.0605	5.75	2.29

For EMG and EEG models the HW column shows the number of the half-wave. For the ECG model the HW column indicates the name of the ECG component considered as a half-wave. Parameter τ indicates the time from which the corresponding HWF starts.

Using defined parameters, it was important to satisfy the condition of Eq (1) by the choice of a sufficiently small time interval Δ during which the expected change of the population size by more than one particle is negligibly small. Using Monte Carlo simulations for estimation of the numbers of particles crossing the membrane, different values of Δ were tested. In this way the value Δ = 0.0001 ms was selected for the following simulations. The corresponding segmentation points over 80 ms time interval were t_i_ = i∙Δ with *i* taking values from 0 to 800,000.

The transition from the resting to transient condition was simulated as the change of the resting state transition probabilities defined by Eq (21) to the transient state transition probabilities in Eqs (18)–(20). The change occurs in a “smooth” fashion. This means that the sizes of the primary and secondary particle populations developed under the resting conditions serve as initial conditions for the transient regimes.

During the resting conditions (interval from -10 ms to t = 0) the transport of particles between the primary and secondary populations is balanced. The particle numbers fluctuate over the mean value equal to N_0_. The manner in which the transition from the resting to transient conditions contributes to a rapid change of the net charge is due almost entirely to the change of the birth and death rates in the primary particle population. In the general case, the size of the primary population is governed by the complex interplay of the birth and death transition probabilities. The onset of the transient conditions gives rise to both probabilities. Initially, from t = 0 to the time instant indicated by the arrow in Fig 7b, the birth probability prevails over the death probability. In this stage nearly a tenfold increase of the size of the primary population occurs. After the peak, the death probabilities take a progressively larger share. As a result, the size of the primary population declines and returns to the initial condition.

Compared with the primary population, the effect of the transients in the secondary particle population on the net charge is minor and brief. As shown in Fig 7c, at t = 0 the probability of birth in the secondary population drops to zero. This blocks particle transfer from the inside to outside of the cells.

In order to decide whether the mass effects of particle movements are sufficient for a full account of the dynamics of macroscale processes, it is necessary to compare the results of computer simulations with the theory. In agreement with Eq (12), we consider transient conditions starting at t = 0. Since ψ(0) = 0, we use ψ_0_ = ψ_P_(0) = ψ_S_(0) as the initial condition for theoretical solutions. The corresponding initial condition for numerical simulations is

$$X_{\text{P}}\left(0\right)=X_{\text{S}}\left(0\right)={\text{N}}_{0}.$$

Defined ψ_0_ and N_0_ parameters allow us to create dimensionless functions ψ*(t) = ψ(t)/ψ_0_ and $X_{N}^{*}(t)={\text{X}}_{N}(t)/N_{0}.$ After this normalization we can directly compare numerically calculated ${\text{X}}_{\text{N}}^{*}(t)$ with theoretical ψ*(t).

**Fig 8:**
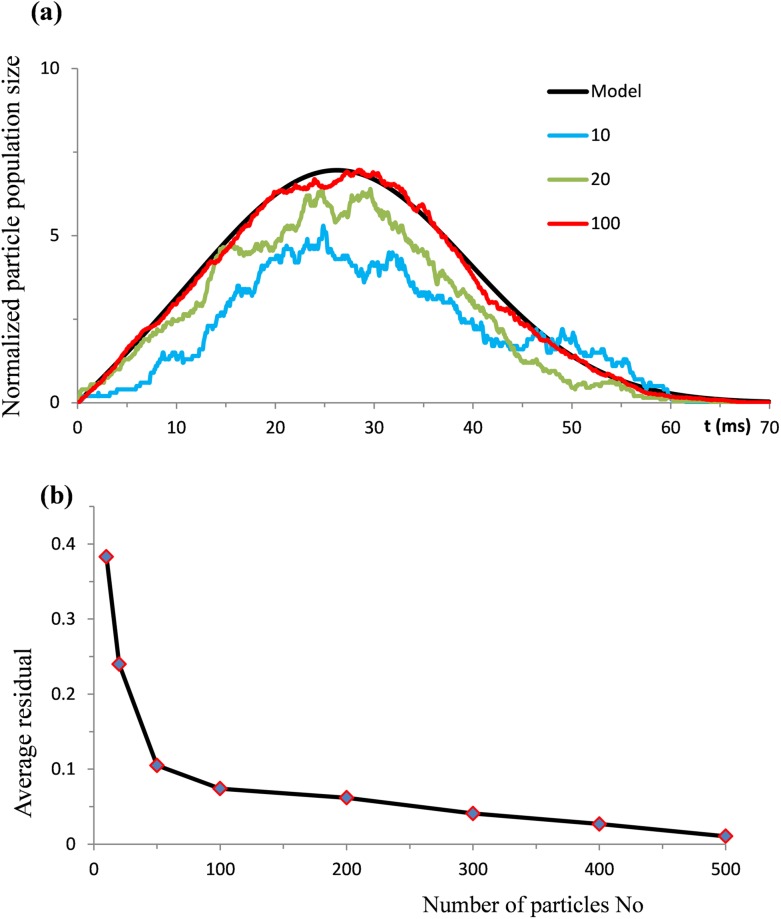
(a) Coloured lines show typical computer reconstructions of the temporal evolution of particle populations with different initial sizes of 10, 20, and 100 particles. The black line is the theoretical solution η*(t). (b) Data points show average residuals between the theoretical and numerical solutions calculated for the particle populations with different initial sizes.

Taking values of σ and β parameters from the previous simulations, the samples of ${\text{X}}_{\text{N}}^{*}(t)$ computed with N_0_ equal to10, 20, and 100 particles are illustrated by the colored lines in Fig 8a.

It is evident that an increase in the number of particles brings single trial samples of ψ*(t) to closer agreement with the theoretical model. Thus, the role of deterministic factors in statistical samples of ${\text{X}}_{\text{N}}^{*}(t)$ increases with an increase in the number of particles involved.

To emphasize this tendency we have estimated absolute values of residuals between ψ*(t) and ${\text{X}}_{\text{N}}^{*}(t)$. Using different values of N_0_ from10 to 500, 20 single trials for each value were calculated. In each trial the residuals were estimated in 100 equidistant points. From these measurements the average residuals were calculated. The points in the plot in Fig 8b show the dependency of average residuals on the number of particles N_0_.

The increase of the particle numbers to several hundreds makes single trials perfectly identical to the theoretical solution. This provides convincing evidence that, under transient conditions, the particle behavior in both primary and secondary particle populations develops as an amalgamation of deterministic and stochastic processes. These are two broadly defined classes of processes with specific properties. We now consider this situation from the point of view of deterministic chaos, i.e. processes that share attributes with both deterministic signals and stochastic processes.

### Transient deterministic chaos

The research on chaos phenomena is progressing steadily, extending to a diverse range of applications. However, in the face of evolving hypotheses and concepts no universal definition of the system producing chaos has been accepted. The necessary conditions are the non-linearity of the system generating chaos and its sensitivity to initial conditions.

A specific of biological systems is that, in most cases, the equations of the sources of deterministic chaos are unknown [24]. Accordingly, the identification of chaotic behavior is usually based on the analysis of empirical time series extracted from the process in question. The most popular methods for characterizing chaotic behaviors are Lyapunov exponents and Kolmogorov-Sinai entropy. These are essentially indirect methods which do not define the equations but diagnose whether the process in question is chaotic or non-chaotic. This limitation applies equally to other diagnostic methods, among which are the Poincare map, correlation dimensions, fractal dimensions, and attractor reconstructions from the time series.

Our approach actually does not need such indirect methods because the theory provides complete models of the HWF generation on both the macro- and micro-scales. The two major criteria which allow us to qualify the system producing these processes as chaotic are as follows.

1. The non-linearity of the system producing HWF.
2. Sensitive dependence of the behavior of the system on initial conditions.

The nonlinearity of the system is evident on the macroscopic scale, where the nonlinear Eq (13) define the temporal evolution of HWF. The chaotic behavior is hidden in the dynamics of mass potentials and is only recognizable by specific Gaussian profiles of the HWF shapes. It is important to note that the specific statistical background of such patterns provided means to establish the rules 1 and 2 that govern chaotic phenomena taking place on the microscale. An important additional factor for confirmation of chaos is that the chaotic system defined by our theory arises solely from the equations of the system, without the need for additional factors.

**Fig 9:**
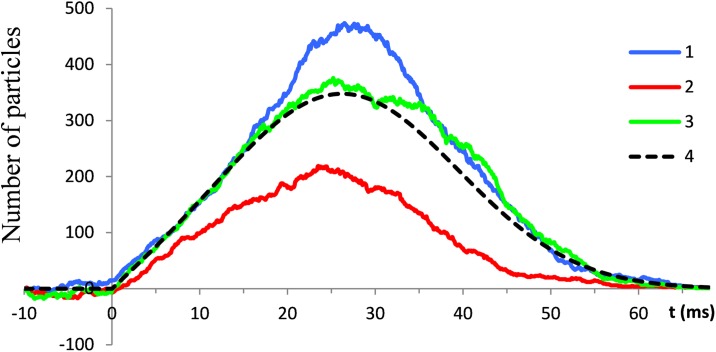
Using the same conditions as the simulations in Fig 8, illustrates the sensitive dependence of X_N_(t) realizations in different trials (coloured lines) on initial conditions. The dotted line is a theoretical solution.

The strong dependency of the chaotic processes on initial conditions is seen from the computer simulations illustrated in Fig 9. The parameters are the same as in the simulations shown in Fig 7. The simulations started 10 ms before arrival of the triggering event. At that instant both P and S populations contained 50 particles. Because the sizes of P and S populations were changing randomly as simple BDPs in the initial 10 ms interval, the initial sizes of the whole population were slightly different in different trials. As the results of the simulations indicate, these minor differences in the initial conditions lead to significantly different future behavior of the realizations of **X**_N_(t).

Another important feature of chaotic processes is short term predictability. It is clear from the simulation results in Fig 9 that, despite substantial differences in the trajectories of **X**_N_(t) realizations, it is possible to give rough estimates of some parameters, for example the times at which the trajectories reach maximums. We may also claim that features of predictability are also incorporated in the time courses of **X**_N_(t) realizations, because the averages of these trajectories from different trials converge on the analytical solutions.

An advantage of the analytical methods of our theory is the possibility to make a clear distinction between the chaos and pure stochasticity. During the resting periods we deal with purely stochastic processes. The start of the HWF generation is specified by the time instant when the triggering event arrives. Thus the deterministic chaos develops as a transient process. We label this amalgamation of deterministic and stochastic processes “transient deterministic chaos”.

### Statistical self-similarity of half waves

An essential outcome of our numerical simulations is the evidence of common statistical and deterministic rules that govern the generation of functional components of mass potentials from different ensembles of multiple excitable cells. This means that cellular ensembles may be divided into constituent parts governed by the same probabilistic and deterministic rules as the whole ensemble. In a most general context, this result may be associated with Mandelbrot’s concept of a fractal, an object composed of similar sub-units that resemble the structure of the whole object [25].

Fractal properties are divided into different categories. Our results may be attributed to the fractal notion of statistical self-similarity. We summarize this outcome of our theory in the following statement of the statistical self-similarity of the half-waves of mass potentials: Consider HWF ψ(t) as a transient mass potential produced by a synchronous activation of a large ensemble L of closely located and functionally linked excitable cells. Let us extract a part of L regarded as a fraction F. We state that the macroscale mass potentials produced by F are governed by the same statistical distribution as the whole population L.

At the macroscopic scale, where statistical factors are hidden, the mass potential develops as the succession of half-wave functions induced at τ_0_,…τ_i,…,_τ_N_ time instants. Thus, the model of a mass potential e(t) has the following form

$$\text{e}\left(t\right)=\kappa_{i}\cdot \sum_{i=0}^{N}\psi_{\text{i}}\left(t-\tau_{i}\right),$$ (22)

where index “i” labels different half-wave functions with corresponding σ and β parameters.

**Fig 10:**
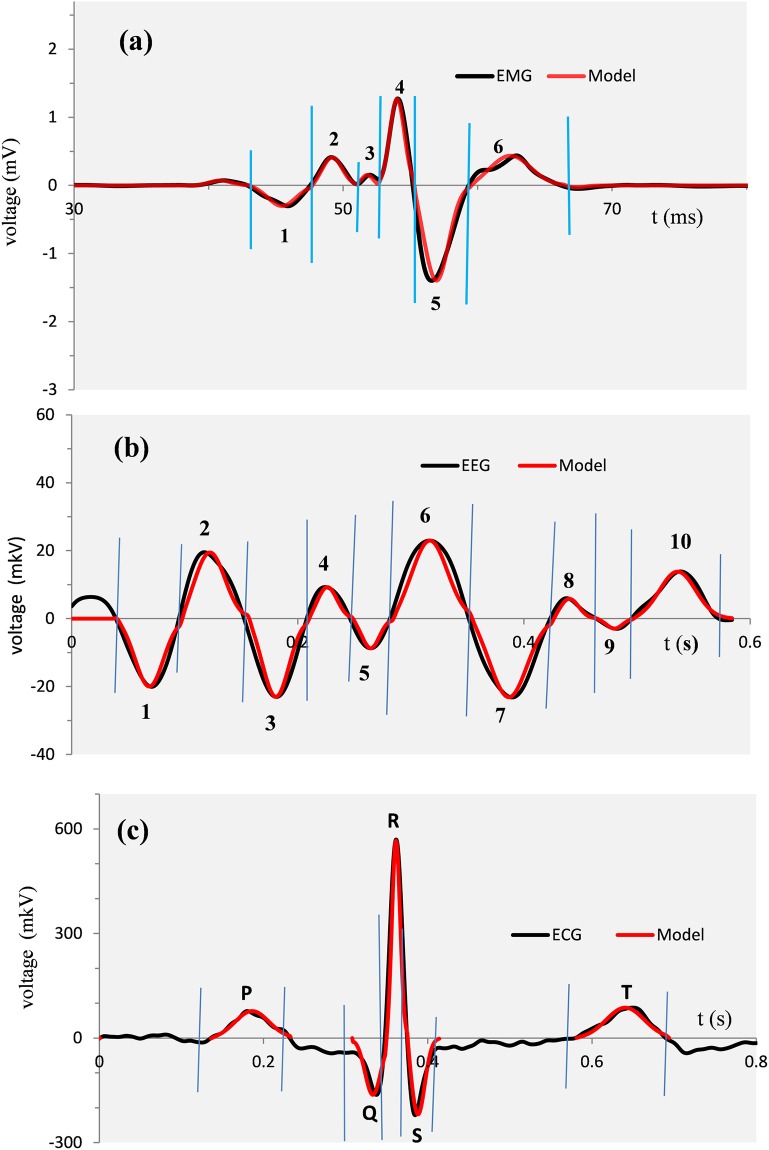
Models of typical mass potentials with parameters τ, κ, σ, and β listed in Table 1. (a, b) Black lines show typical records of eye-blink EMG and EEG. Vertical blue lines indicate the segmentation points. The red lines are the models calculated from Eq (22) as the sums of models created for each empirical half-wave (signal fragment between adjacent segmentation points). (c) The same illustration of results as in (a) and (b), except that models are only shown for P, Q, R, S, and T ECG components.

Characteristic examples of the models of different mass potentials are shown in Fig 10.

The high accuracy of Eq (22) is a remarkable outcome of our theory since the HWFs building the models were derived entirely from experimental records without any adjustments to various origins of the mass potentials to which the models were applied.

Table 1 lists the values of parameters supporting the models in Fig 10.

## Discussion

Quantum mechanics provides successful explanations of microscopic phenomena in almost all branches of physics, and also serves as an indispensable source of guiding principles for efforts to create quantum biology [26–28] and, particularly, quantum neurobiology [29].

Quantal analyses play a vital role in understanding the junctional mechanisms by which information is transmitted in the nervous system, from cell to cell across synapses. In the early 1950s, Bernard Katz and his colleagues revolutionized synaptic physiology with the discovery that transmitter substance is released from a terminal in the form of acetylcholine packets of a constant size [30]. This achievement earned Katz the Nobel Prize for Physiology and Medicine in 1970, and established the concept of the acetylcholine quantum as a fundamental entity of transmitter substance. Various models of different synaptic phenomena have been created on this probabilistic basis.

The first author of the present paper (DM) has developed a double barrier quantal model of neurotransmitter release in which the changing amounts of quanta available for release have been deduced as the result of quanta turnover between the two postulated presynaptic pools of transmitter [11, 12]. The quanta exchange between the pools has been described in the probabilistic terms of a linear birth-and-death process (BDP).

In the present study we preserve the general principles of this modelling approach but employ a more wide-ranging class of Markov processes, non-homogenous BDP. This probabilistic framework provides a radically different approach to the analysis and interpretation of mass potentials than did previous deterministic models of mass potentials.

The major assumption of deterministic models is that dynamics of the mass potential can be deduced as the superposition of membrane potentials produced by the underlying cellular elements. A general mathematical framework is known as volume conductor theory, several versions of which address EEG [31], ECG [15], and EMG [32]. Significant difficulties are created by the intractably huge multiplicity of cellular elements, along with an insufficient knowledge of their parameters. Under such uncertainty little confidence can be placed on particular solutions from a large number of different models.

The idea of an alternative phenomenological approach using statistical considerations has been put forward by Elul [33]. Based on the analysis of the synchronization of EEG sources, Elul proposed that evolution of brain waves may be governed by statistical regularities following from the central limit theorem. Thus, EEG may simply be accounted for as the normally distributed output resulting from a combination of the activity of many independent neuronal generators.

The mathematics behind these propositions has never been worked out in detail. The major problem is to support probabilistic propositions by a quantitative microscale model. That is, the combination of general probabilistic methods with specific particle models create the tools of the probabilistic formalism of quantum mechanics, with unique power to link the microscale and macroscale processes.

As far as we are aware, our study is the first to provide an empirically grounded microscale model of mass potentials, and the first to approach the genesis of mass potentials on this original probabilistic basis. This permits a great deal to be inferred about the mass effects of very complicated cellular structures without even mentioning cells and channels, or even being very explicit about internal makeup. A fundamental point is the consideration of deterministic components of mass potentials as a limit of underlying microscale processes. The crucial step is the change of basic microscale units from continuous time membrane potentials to elementary particles (point charges). The vanishingly small role of individual particles in the generation of the macroscopic scale processes reduces the problem to the study of the limiting behavior of large numbers of random variables. This is a classical problem in probability theory [34]. Some purely mathematical aspects of how deterministic potentials, such as membrane potentials, emerge as a limit from the underlying stochastic sources have been recently considered by Austin [35].

Referring to various probabilistic models of ion transport [36], the new feature of the probabilistic framework of our theory is the implementation of non-homogenous BDPs with time dependent rates of the birth and death. There are at least two important consequences of this extension of the probabilistic tools employed.

First, it becomes possible to extend purely stochastic models of microscale machinery by emergence of transients with deterministic trends, which we have classified as transient deterministic chaos.

Second, the link was established by Eq (6) between the microscale transients and macroscale components of mass potentials. This link provides a way to estimate the microscale parameters on an empirical basis by consideration of mass-potentials as non-stationary processes.

On the macroscopic scale we use fragmentary decomposition, a model-based method of non-stationary signal analysis, to decompose mass potentials into functionally meaningful components on an adaptive segmentation basis [16, 17]. At this step we follow the conventional empirical understanding of functional components as the positive and negative peaking waveforms that are observed in the time course of the mass potential. The possibility to transfer these empirical pieces of mass potentials to the universal analytical model has been originally indicated by the results of the time-frequency analysis of the late component waveforms (N_1_, P_2_, N_2,_ and P_3_) of ERPs recorded in a conventional auditory oddball paradigm [37]. It appeared that, although different components are associated with different neural sources, their normalized amplitude spectra are practically identical, being accurately described over a wide range of frequencies by a Gaussian function. Similar results have also been obtained for single trial eye-blink EMGs [16] and conventional P, Q, R, S, and T components of ECG [38]. This study unites previous considerations of different mass potentials into a general quantum theory which supports the reconstruction of various mass potentials on a common theoretical and computational basis.

The striking similarity of the amplitude spectra of different mass potentials can be seen by comparing the plots in Fig 1c and the lower panel of Fig 3. Such results are somewhat difficult to reconcile with our physiological intuitions and concepts about different structure and functions of EEG, ECG, and EMG generators. To provide a clue to the understanding of this seemingly paradoxical situation, we note that transition from a deterministic to a quantum model replaces consideration of physical parameters of the underlying sources with an account of their statistical properties. In this context, an elementary source of electricity is a point charge carried by an extracellular ion and treated as a random variable. The mass effect emerges as a limit from the collective behavior of a large number of point charges acting on the microscopic scale. The appearance of Gaussian functions in the basic Eqs (9) and (12) indicates that the mass effect produced by multiple microscale events is governed by the rules of the central limit theorem. The theorem states that any process of random sampling, given a sufficiently large sample, tends to produce a normal distribution of sample values, even if the subsets of the whole population from which the samples are drawn do not follow a normal distribution. An important aspect of this phenomenon is that statistical regularities considered by the central limit theorem are independent of the physical nature of the objects from which the statistical samples are drawn. Thus, we may explain the resemblance of the amplitude spectra of EEG, ECG, and EMG signals by the fact that, although the cellular organization of these signals is quite different, the molecular events on the microscopic scale are governed by similar statistical regularities.

Applications of the central limit theorem are usually associated with single normal distribution. In such cases the sources of random samples are stationary stochastic processes.

The ability of our theory to deal with transients is provided by the specific composition of the HWF from two Gaussian functions with identical parameters. We regard these Gaussian functions as products of separate but operationally linked particle populations with identical parameters. The primary population contains particles of the same polarity as the macroscopic component. The particles from the secondary population have an opposite polarity. Thus, an important finding of our study is that, during development of the transient deterministic chaos on the microscopic scale, the two sorts of participating particles with opposite polarities are recognizable from the macroscopic scale.

We may state that HWF ψ(t), emerging as the macroscopic scale effect from synchronized chaotic ion movements on the microscopic scale, can be regarded as a universal building block from which various mass potentials are composed. In terms of quantum mechanics, this effect can be qualified as *universality*, the evidence that there are properties for a large class of systems that are independent of the exact structural and functional details of the system. This probabilistic basis implemented by our theory provides the means to reduce intractably huge numbers of microscale variables to a universal macroscale object in the form of a HWF with the fewest parameters (κ, σ, and β). As limit distributions for the sums of random variables, these parameters discriminate those aspects of the molecular machinery that are significant on the global scale from those that are not.

The possibility to decompose a complex signal into a set of universal “building blocks” plays an important role in different fields of physics and biology. Thus, synaptic physiology has greatly benefited from the quantum analysis which deduced a miniature end potential as the building block from which postsynaptic potentials are composed. An important outcome is that this concept is applicable to various types of synaptic junctions with different molecular organizations. This is a remarkable feature of quantal universality which applies equally to our treatments.

To our knowledge, our study is the first to put the models of various types of mass potentials on a common theoretical and computational basis. Using HWF as a universal building block, we obtain a general model of the mass potential in the form of Eq (22). This equation suggests that a mass potential evolves as the succession of transient components induced at consecutive time instants. Each component is a HWF described by the system of non-linear Eq (13) with specific sets of estimated parameters.

Conventional procedures of parameter estimation consider a mass potential as the set of components in the form of peaking waves. Given this widely accepted conceptual framework, the variety of different scoring engines, known as peak picking procedures, reduce an electrophysiological signal to the peak amplitudes and latencies. A critical limitation of these procedures is that measurements of a signal at isolated time points are unable to characterize the waveform dynamics which contains important functional and diagnostic information.

Considering the component as being not just a peak in the waveform, but a whole deflection (positive or negative) with a specific shape, the HWF is an adequate analytical model of such an entity. The timing (τ) and magnitude (κ) of a HWF correspond to conventional component parameters. However, no parameters have so far been accepted as adequate measures of the component shapes. In this regard, the σ and β parameters appear as universal shape parameters with the potential to provide important information about the dynamics of the underlying microscale processes. The positive impact of this innovation would be to open up new ways of modeling the mass potentials in both physical and physiological settings. The flexibility and remarkable accuracy of this methodology is seen in Fig 10, where excellent fits to the samples of different mass potentials (EEG, ECG, and eye-blink EMG) with various activity patterns have been obtained using HWF as a universal component of the dynamic model.

The possibility to decompose different sections of mass potentials from different spatial locations into ensembles of universal building blocks identifiable on an empirical basis may have important applications for solutions to the forward and inverse problems in electrophysiology. The uniqueness and universality of the HWF provides a means to avoid unsubstantiated speculations about physical factors contributing to the generation of mass potentials. However, the aims of these problems, particularly predictions of the spatial locations of mass potentials, are quite specific and demand special considerations. We are planning to approach some of these problems in future papers.

## Materials and methods

### Time-frequency analysis using the SBF algorithm

The time-frequency analysis is applied to the time series of a mass potential presented by Eq (7) as the sum of empirical HWs. It is necessary to transform each w_i_(t) to the frequency domain using numerical calculation of the exponential finite Fourier transform expressed by Eq (8). Procedures applied to various HWs are universal. Thus, we omit subscripts in HW descriptions and consider w(t) as a target for the following transformations. The corresponding complex spectrum is defined by the following Fourier transform

$$W(i\omega )=\int_{0}^{T}w(t)\text{exp}(-i\omega t)dt.$$

Numerical estimation of Fourier transforms is usually based on procedures employing various algorithms of the fast Fourier transform. The latter is supported by a Fourier series model of the data, i.e. the addressees are periodic signals [39]. This distinction with the Fourier integrals is a troublesome problem when aperiodic signals of short duration, like HW, are analyzed. When a waveform is not periodic in time, the spectral leakage may cause significant distortions of the frequency characteristics.The remedies of windowing and zero-padding usually introduce problems of their own.

As an adequate tool of the spectral analysis of HWs we use the SBF algorithm [18]. The algorithm is an original version of Filon-type methods that provide maximum precision in the estimation of trigonometric integrals using interpolation polynomials of different degrees [40].

Using the SBF algorithm we deal with numerical calculations of the real and imaginary parts of the complex spectrum W(iω):

$${\text{W}}_{C}(\omega )=Re[\text{W}(i\omega )]=\int_{0}^{T}\text{w}(t)\text{cos}(\omega t)dt,$$

$${\text{W}}_{S}(\omega )=Im[\text{W}(i\omega )]=\int_{0}^{T}\text{w}(t)\text{sin}(\omega t)dt,$$

where W_C_(ω) and W_S_(ω) are finite cosine and sine Fourier transforms of w(t) and [0, T] is the interval of integration.

For numerical calculations w(t) is specified by its sampled values w_i_ = w(t_i_), where t_i_ = iΔ, i takes values from 0 to N and t_N_ = T. Over these samples a piece-wise linear polynomial h(t) is created.

**Fig 11:**
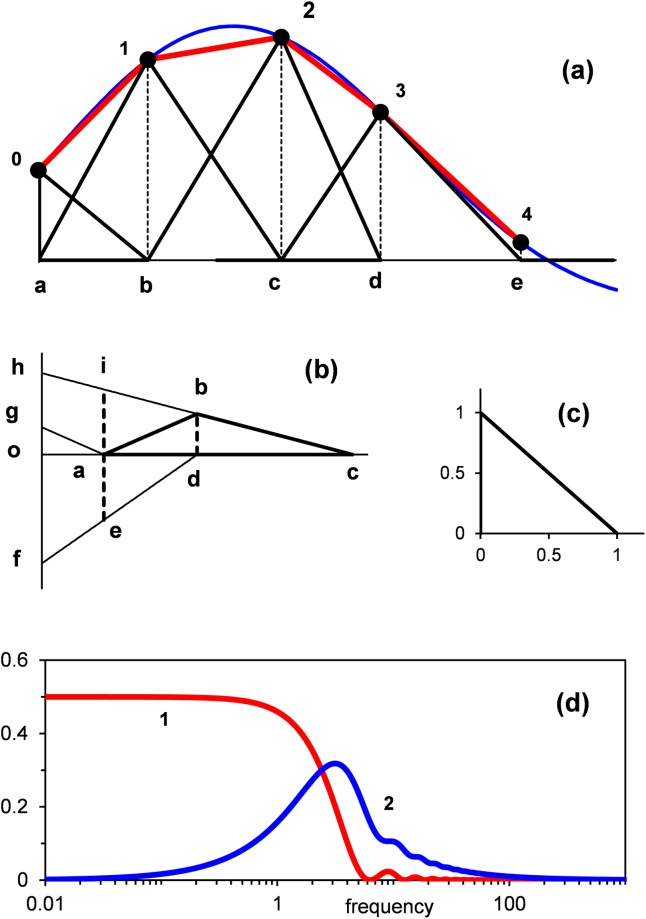
(a) Exemplifies a piece-wise linear interpolation of half-wave function. (b) Shows construction of the hat function (triangle **abc**) as the sum of the triangles **ohc**, **ofd** and **oga**. (c) The triangular basis function. (d) The curves 1 and 2 show functions (26) and (27), respectively.

Fig 11(a) illustrates the principle of piecewise-linear interpolation. Given w(t) (blue line), the interpolant, h(t), is the broken red line created by joining the nodal points 0, 1, 2, 3 and 4 by the straight lines. In the same fashion the interpolation can be continued for any number of the following nodal points.

A specific aspect of the SBF algorithm is that h(t) is decomposed into the weighted sum of similar basis functions of the following form

$$\text{h}\left(t\right)=\sum_{i=0}^{N-1}a_{i}r\left(t/t_{i+1}\right),$$ (23)

where a_i_ is the interpolation coefficient and r(t) is a similar basis function defined in the form of the triangular basis function (TBF):

$$\text{r}\left(t\right)=\{\begin{array}{ll} 1-t & \text{if}\;\;0\leq t\leq 1\\ 0 & \text{otherwise} \end{array}$$

The simple geometric form of TBF (unit right-angled triangle) is illustrated in Fig 11c.

The interpolant, h(t), h(t) is created as a piece-wise linear polynomial which satisfies the interpolation condition:

$$h_{i}=w_{i}\;\text{for}\;\text{i}=0,1,\ldots ,\text{N}-1,$$ (24)

where *h*_*i*_ = *h*(*t*_*i*_).

A general procedure of the evaluation of the interpolation coefficients consists of writing Eq (23) for each interpolation point and finding the solution of the resulting system of N linear equations by conventional matrix methods [18].

While this set of linear equations is easily solved by standard methods, there is a further simplification possible due to the fact that *r*(*t*_*m*_/*t*_*n*_) = 0 for m≥n. To make this mode of interpolation as intuitive as possible, we use as an intermediate element the hat function, one of the geometrical objects employed by the method of finite-elements [41]. The hat function is defined as

$$\vartheta_{i}\left(t\right)=\{\begin{array}{ll} \frac{t-t_{i-1}}{t_{i}-t_{i-1}}, & \text{if}\;\;t_{i-1}\leq t<t_{i}\\ \frac{t_{i+1}-t}{t_{i+1}-t_{i}}, & \text{if}\;t_{i}\leq t<t_{i+1}\\ 0, & \text{otherwise} \end{array}$$

on the mesh t_0_<t_1_<…<t_i_<…<t_N_.

Triangles **a1c**, **b2d**, **c3e** in Fig 11a and **abc** in Fig 11b are examples of weighted hat functions.

The interpolation capability of the hat function is supported by the two properties. First, ϑ_i_(t) vanishes everywhere except on the two subintervals to which t_i_ belongs. Second, ϑ_i_(t) is unity at the node i and zero at all other nodes.

Using the hat function, the interpolant (23) may be presented in the form

$$\text{h}\left(t\right)=w_{0}r\left(t/t_{1}\right)+\sum_{i=1}^{N-1}w_{i}\vartheta_{i}\left(t\right).$$ (25)

Now the interpolation coefficients are equal to the samples of w(t). This simplification is the result of the change of the basis function from the TBF in Eq (23) to the hat function in Eq (25). The geometric principle found useful in this connection may be seen by reference to Fig 11. Each nodal point from 0 to 3 in Fig 11a is linked with a particular triangle. The nodal point 0 is the top of the right-angled triangle which corresponds to the first terms in Eqs (23) and (25). The nodal points from 1 to 3 are the tops of the hat functions multiplied by the interpolation coefficients (triangles **a1c**, **b2d** and **c3e**). The graph in Fig 11b shows that the hat function (triangle **abc** with **bd** = 1) may be decomposed into the sum of three TBFs triangles **ohc**, **ofd** and **oga**). Thus, in the interval from t_i-1_ to t_i+1_ the corresponding hat function is defined as

$$\vartheta_{i}(t)=\alpha_{i}r(t/t_{i+1})-\beta_{i}r(t/t_{i})+\gamma_{i}r(t/t_{i-1})$$

where

$$\begin{array}{l} \alpha_{i}=\frac{t_{i+1}}{\Delta t_{i+1}}\;\;\;\left(0\leq i\leq N-1\right),\\ \beta_{i}=t_{i}\frac{\Delta t_{i+1}+\Delta t_{i}}{\Delta t_{i+1}\Delta t_{i}}\;\;\;\left(1\leq i\leq N-1\right),\\ \gamma_{i}=\frac{t_{i-1}}{\Delta t_{i}}\;\;\;\left(2\leq i\leq N-1\right), \end{array}$$

and Δt_i_ = t_i_-t_i-1_.

In these terms

$$h(t)=w_{0\;}r(t/t_{1})+w_{1}[\alpha_{1}r(t/t_{2})]-\beta_{1\;}[r(t/t_{1})]+\sum_{i=2}^{N-1}w_{i}\vartheta_{i}(t).$$

Comparison with Eq (23) shows that interpolation coefficients are readily found to be

$$\begin{array}{cc} {\text{a}}_{i}=\alpha_{i}w_{i}-\beta_{i+1}w_{i+1}+\gamma_{i+2}w_{i+2} & \left(i=0,\ldots ,N-1\right), \end{array}$$

where α_0_ = 1 and it is assumed that w_o_ = 0 and *w*_*N*+1_ = 0.

The system of simultaneous linear equation has thus been reduced to a set of independent linear equations. Now h(t) is described in terms of a linear combination of TBFs, the finite Fourier cosine- and sine- integrals of which are given by

$${\text{R}}_{C}\left(\omega \right)=\int_{0}^{1}\text{r}\left(t\right)\text{cos}\left(\omega t\right)dt=\frac{1-\text{cos}\left(\omega \right)}{\omega^{2}},$$ (26)

$${\text{R}}_{S}\left(\omega \right)=\int_{0}^{1}\text{r}\left(t\right)\text{sin}\left(\omega t\right)dt=\frac{\omega -\text{sin}\left(\omega \right)}{\omega^{2}}.$$ (27)

These functions are illustrated in Fig 11d.

According to the similarity theorem of the theory of Fourier transforms, the compression of the abscissa in the time domain corresponds to the expansion of the abscissa plus contraction of the ordinate in the frequency domain. These relationships reduce the entire issue of the transform calculations to some standard manipulations with relatively simple functions R_C_(ω) and R_S_(ω). The cosine- and sine- Fourier integrals from h(t) obtain the following representations

$$\begin{array}{l} {\text{W}}_{C}\left(\omega \right)=\sum_{i=0}^{N-1}a_{i}t_{i+1}\frac{1-\text{cos}\left(t_{i+1}\omega \right)}{{\left(t_{i+1}\omega \right)}^{2}},\\ {\text{W}}_{\text{S}}\left(\omega \right)=\sum_{i=0}^{N-1}a_{i}t_{i+1}\frac{\omega t_{i+1}-\text{sin}\left(t_{i+1}\omega \right)}{{\left(t_{i+1}\omega \right)}^{2}}. \end{array}$$

The corresponding amplitude spectrum W(ω) and phase function δ(ω) are defined as:

$$\text{W}(\omega )=|\text{W}(i\omega )|=\sqrt{{\text{W}}_{C}^{2}(\omega )+{\text{W}}_{S}^{2}(\omega ),}$$

$$\delta (\omega )=\text{arctg}[W_{S}\left(\omega \right)/W_{C}(\omega )].$$

These are continuous functions of angular frequency. Accordingly, the values of the frequency domain characteristics can be calculated for arbitrary sets of points. We employ a logarithmic frequency scale for calculations of amplitude spectra and a natural frequency scale for calculations of phase functions.

In the case of a logarithmic scale, the frequency characteristics were calculated for angular frequencies ω_i_ = ω_0_C^i-1^ (i = 1,…,N), where ω_0_ is initial angular frequency and C>1 is the parameter that defines the sampling rate. For selection of this parameter it is convenient to use the formula C = exp(ln10/N_D_), where N_D_ is the number of samples per decade. In the case of a natural frequency scale, ω_i_ = ω_0_+iΔω (i = 0,…,N), where Δω is the discretization step.

### Kolmogorov Smirnov test

An important prerequisite of the statistical analysis is that the empirical amplitude spectra that we deal with are normalized for both amplitude and frequency. These are dimensionless theoretical and empirical spectra, G(γ) and Z(γ) respectively.

For comparison of these spectra we refer to the discrete values of relative frequency γ denoted as γ[i] = γ_0_C^i-1^ (i = 1,2,…) where γ_0_ = ω_0_/ω_C_. These are evenly distributed points on the logarithmic abscissa scale.

To estimate discrepancy between G(γ) and Z(γ) we measure MSE[m,n], which represents the mean square error calculated at the range of frequencies γ[i] from i = m to i = n.

Observations of various amplitude spectra revealed that, typically, the fits virtually coincide in the range of relative frequencies from 0 to 1. At γ>1 the errors increase with increase of frequency. Taking into account these peculiarities, we organized the tests of discrepancies as a two-step procedure using empirically established error thresholds T1 = 0.0001 and T2 = 0.002.

1. The range of relative frequencies from γ[0] to γ[J] = 1 is selected. It corresponds to the frequency band from F_0_ = ω_0_/2π to F_C_. The fit is accepted and followed by step 2 if MSE[0,J]<T1.
2. Starting from i = 1 the MSE[J+i-2,J+i+3] is calculated for increasing numbers of i until MSE[J+i-2,J+i+3]>T2. Thus, we have a six point window which moves from F_C_ to the higher frequencies. The point m = i at which the procedure stops defines the boundary frequency F_B_ = γ[m]∙F_C_. Thus, an acceptable fit extends over the frequency range from F_0_ to F_B_. The corresponding dimensionless extension ratio ε = F_B_/F_C_.

Given the samples of ε in the form of two different ensembles, $E_{1}=\{\varepsilon_{1,\;}^{1},\;..,\varepsilon_{n}^{1},..,\varepsilon_{N}^{1}\}$ and $E_{2}=\{\varepsilon_{1,\;}^{2},\;..,\varepsilon_{k}^{2},..,\varepsilon_{K}^{2}\}$, we use the Kolmogorov-Smirnov two sample test in order to decide whether $E_{1}$ and $E_{2}$ are produced by the same or different distributions [42]. Each of the data sets $E_{1}$ and $E_{2}$ is converted to the cumulative frequency distribution. The test is based on the evaluation of the maximum vertical deviation D between the cumulative frequency distributions. The null hypothesis that the two distributions are the same is rejected if the value of D exceeds the critical value defined by the tables of D statistics.

### Algorithm of numerical simulations

The processes to be reconstructed in numerical simulations are the BDPs satisfying the general condition in the form of Eq (1). Specific aspects of particle turnover in the primary and secondary particle populations are taken into account by empirically based transition probabilities defined by Eqs (18)–(21). Calculations are organized as the succession of standard steps dealing in sequential order with the time intervals [t_i_, t_i+1_] for i taking values from -M to N-1. The time interval from t_-M_ to t_0_ corresponds to the resting conditions. At t = t_0_ the resting state is switched to the transient conditions.

For the primary particle population the procedure for each step is as follows:

1. Set x_i_ to be the initial state of the particle population. For the first interval beginning at t_-M_ define the initial state by an arbitrary integer N_0_, i.e. x_-M_ = N_0_.
2. Estimate the transient probabilities from Eqs (18) and (19) for the transient conditions, or use Eq (21) for the resting conditions.
3. Pick out random real numbers **r**_b_ and **r**_d_ using a random number generator to produce real numbers in the range from 0 to 1.
4. Estimate the size of the particle population at the end of the interval x_i+1_ = x_i_ + *b* − *d*, where *b* and *d* are the binary numbers defined as follows.

**Resting conditions:**
b = 1 if **r**_b_<p_r_(i) and is zero otherwise,
d = 1 if **r**_d_<p_r_(i) and is zero otherwise.

**Transient conditions:**
*b* = 1 if $r_{b}<{\hat{p}}_{P}(i)\;$ and is zero otherwise,
*d =* 1 if $r_{d}<{\overset{ˇ}{p}}_{p}(i)\;$ and is zero otherwise.

For the secondary particle population the procedures are similar, with the exception that components of the birth process under the transient conditions are excluded from consideration.

### Empirical mass potentials: EEG, ECG, eye-blink EMG

A powerful element of probabilistic methods of quantum mechanics is universality, an ability to discriminate macroscale properties that are independent of the exact structural and functional details of an underlying microscale system [43]. To be able to deal with this aspect of our theory on an empirical basis we have selected EEG, ECG, and eye-blink EMG mass potentials. Although the cellular origins of these signals are quite different, they all depend upon ions crossing a membrane, leaving one compartment and entering another.

As characteristic samples of EEG, ECG, and eye-blink EMG signals, we used the data available from previously published studies [37, 44, 45]. Ethical approval was provided by the Macquarie University Human Research Ethics Committee (Medical Sciences) (EC00448) and by the Wake Forest University Institutional Review Board (IRB00021587).

Though there is some uncertainty with regard to the generators of EEG, there is general agreement that EEG arises from synchronized synaptic activity in populations of cortical neurons [46]. Also, the non-excitable glial cells have been shown to contribute to these processes, especially at low frequencies [47, 48].

The EEG data used in our analyses were collected and described in a study of single-trial auditory event related potentials [37]. A standard auditory “oddball” paradigm was employed. The EEGs were recorded from 40 healthy subjects (20 males and 20 females; age 20–50 years). Stereo headphones conveyed in a random order 1500-Hz task-relevant tones and 1000-Hz task-irrelevant tones. EEGs were recorded from 19 electrode sites according to the 10–20 international system and off-line electro-oculogram artefact corrected using a technique based on Gratton et al. [49]. The EEG segments for analysis have been extracted from digitally stored data sets from both pre- and post- stimuli intervals.

The surface ECG, obtained by recording the potential differences between electrodes placed on the surface of the skin, is a common indicator of electrical activity of the heart, both in clinical and research settings. Conventional conceptualization of the ECG as an ensemble of P, Q, R, S, and T major waves associates these deflections in ECG with different aspects of heart performance during the cardiac cycle. The P-wave reflects the excitation of the atria, the QRS complex that of the ventricles, and the T-wave is associated with the recovery of the initial electrical state of the ventricles. This means that different microscale events produce half-waves identified in the ECG time course.

The ECG data used in our analyses were collected and described in a study of the dynamics of ECG waveforms [44]. For purposes of this study, the ECG data from a group of 14 healthy subjects (7 males and 7 females; age 22–62 years) were selected. Utilizing built-in selection facilities of an ELI 250c Mortara electrocardiograph, standard 10 s segments of filtered (0.05 to 300 Hz frequency range) and digitized (1 ms sampling rate) ECGs were extracted from each of 12 leads for the time-frequency analysis.

Because the eye-blink EMG is less familiar to a wide audience than EEG and ECG, we provide some basic facts about this remarkable electrophysiological signal. Eye-blink EMG is an indicator of motor unit action potentials in the orbicularis oculi muscle that are caused by activity of the facial nerve (CN7) [50]. It is widely used as an electrophysiological marker of the startle reflex, i.e. a brainstem response to a sudden stimulus, such as a sound, a flash of light, a tap to the forehead, a puff of air to the side of the face, or an electrical pulse to the forehead [51]. Enabling the evaluation of information processing at different levels of the central nervous system, eye-blink EMG has been used in a wide variety of research and clinical applications in humans, to study basic stimulus processing [52, 53], attentional factors [54], emotion [55], and personality variables [56].

Eye-blink EMG is measured from surface electrodes placed on the skin overlaying the orbicularis oculi, and develops over time as a succession of positive and negative deflections (peaks) generally accepted as physiologically and clinically meaningful components.

The results are from a study of 35 healthy undergraduate students (ages 18–22 years). Startle eyeblinks were elicited with a 50 ms duration 100 dB broadband noise with an instant rise time, presented via Sennheiser headphones. EMG data were collected using 4 mm inner diameter biopotential electrodes attached to the face below the left eye. The EMG signal was filtered by a Biopac EMG 100 bioamplifier, passing 0.1 to 500 Hz, and was sampled by a Biopac MP150 workstation at a rate of 5000 Hz. A multiple-pass moving averaging was further applied for baseline wander correction. After these procedures 300 ms EMG segments were extracted from single trials (50 ms pre-stimulus onset and 250 ms post-stimulus onset). Only data from control startle trials (no prepulse, no task) were included in this analysis.
